# Phage amplification-coupled CRISPR/Cas12a system for selective detection of viable *E. coli* in fresh produce

**DOI:** 10.3389/fmicb.2026.1770383

**Published:** 2026-04-01

**Authors:** Nicharee Wisuthiphaet, Ahmed El-Moghazy, Nitin Nitin

**Affiliations:** 1 Department of Biotechnology, Faculty of Applied Science, King Mongkut’s University of Technology North Bangkok, Bangkok, Thailand; 2 Department of Microbiology and Plant Pathology, University of California, Riverside, Riverside, CA, United States; 3 Department of Food Science and Technology, University of California, Davis, Davis, CA, United States; 4 Department of Biological and Agricultural Engineering, University of California, Davis, Davis, CA, United States

**Keywords:** bacterial detection, bacteriophage, CRISPR/Cas12a, *E. coli*, food safety

## Abstract

Rapid and specific detection of viable foodborne pathogens is critical for ensuring the safety of the food supply chain and preventing the risk of foodborne illness outbreaks. In this study, bacteriophage T7 amplification was integrated with a CRISPR/Cas12a-based assay for detecting viable *E. coli* in model systems, including fresh produce homogenate, using minimal instrumentation and without requiring conventional nucleic acid amplification steps. The CRISPR/Cas12a reaction condition was optimized for the detection of bacteriophage T7 DNA amplification upon infection of viable bacterial cells. The optimal concentrations of Cas12a and crRNA were 250 and 500 nM, respectively, with an optimal isothermal temperature of 50°C. The developed detection approach enabled the detection of viable *E. coli* at concentrations as low as 1 CFU/mL in culture broth and 10^2^ CFU/mL in spinach homogenate within an 8-h-15-min timeframe, without the need for additional DNA amplification. The developed approach exhibited high specificity for *E. coli* detection in the presence of a mixture of non-targeted bacteria. This work highlights the potential of phage-assisted CRISPR/Cas12a systems for rapid, low-cost, and reliable detection of viable foodborne pathogens in complex food matrices.

## 1 Introduction

The rapid and specific detection of live pathogenic bacteria in food production and processing environments is crucial for ensuring the safety of the food supply chain, particularly for minimally processed and ready-to-eat foods (Nnachi et al., 2022). Timely pathogen detection is critical not only to prevent foodborne illnesses but also to minimize economic losses. The traditional culture-based approaches for the detection of bacteria from food samples involve multiple steps, including enrichment, isolation of colonies and biochemical analysis that can require 3–5 days to obtain confirmed results (Feng et al., 2011). Thus, there is a need to develop rapid approaches for the detection of bacteria in food systems, particularly foods with limited shelf life such as fresh produce. To address these challenges, several molecular-based detection technologies have been developed to enhance sensitivity, specificity, and reduce detection time (Kotsiri et al., 2022; Pan et al., 2022; Saravanan et al., 2021). Among these, molecular detection technologies based on amplification of target nucleic acid sequences have been adopted by industry to enhance the specificity and speed of foodborne pathogen detection. Conventional nucleic acid amplification technologies, such as real-time polymerase chain reaction (PCR), rely on non-isothermal amplification processes. While real-time PCR is highly sensitive and specific, its use is somewhat constrained as it requires relatively expensive instrumentation, skilled personnel, and controlled laboratory conditions to isolate and analyze nucleic acids from food and environmental samples (Klein, 2002).

To address some of these technical challenges, isothermal amplification technologies such as recombinase polymerase amplification (RPA), loop-mediated isothermal amplification (LAMP), and others have been developed (Garg et al., 2022; Li et al., 2022; Zhang et al., 2024). These technologies address some of the constraints of PCR-based detection methods by offering simplicity, faster reaction times, and suitability for field applications, as they do not require sophisticated thermal cycling equipment. In addition, isothermal and non-isothermal nucleic acid amplification technologies can be combined with CRISPR/Cas-assisted detection of amplified nucleic acids. In this detection approach, collateral cleavage of quenched reporter nucleic acid probes by the Cas protein generates a fluorescence signal. The collateral cleavage is triggered by the binding of the crRNA to the DNA template and assembly of the CRISPR/Cas complex (Broughton et al., 2020; Li Y. et al., 2019; Li et al., 2018b; Teng et al., 2019). This additional step using CRISPR/Cas could enhance detection sensitivity by secondary amplification of the detection signal after initial amplification of the target nucleic acids. For example, recombinase polymerase amplification (RPA) is followed by transcription of the double-stranded DNA template to generate short single-stranded RNA (70–120 nucleotides long), which is then detected with the CRISPR/Cas13 system (Gootenberg et al., 2017). Similarly, loop-mediated isothermal amplification (LAMP) has been used as a detection technique in conjunction with CRISPR/Cas12b (Li L. et al., 2019) to amplify and detect target nucleic acid sequences.

However, for the detection of target bacteria in food systems, many of the nucleic acid amplification technologies lack the specificity to discriminate the viability of bacterial cells (Li et al., 2022; Xia et al., 2022), as the detection of DNA or RNA alone does not necessarily indicate the presence of live pathogens. As a result, the food industry often uses a combination of culture-based and nucleic acid-based testing approaches to confirm microbial test results, which introduces additional complexity and increases overall operational time for detection (Foddai and Grant, 2017). Overall, there is a need to improve the discrimination between live and dead bacteria in food systems and to reduce the extensive resources required by many nucleic acid-based detection technologies. To address some of these limitations, bacteriophages, viruses that infect bacteria, have been used as powerful tools for bacterial detection due to their high host specificity and rapid amplification upon infection. Since bacteriophages are highly specific to their hosts, they enable bacterial detection without the need for colony isolation, biochemical identification, or post-culture genetic confirmation, which are typically required in culture-based detection approaches, thereby simplifying the workflow and reducing the total turnaround time. In biosensing applications, phages function as biorecognition elements that selectively recognize and infect viable target bacteria (Ahovan et al., 2020). The phages can also act as transducers by generating measurable signals upon infection. These signals may arise from bacterial cell lysis and the consequent release of intracellular enzymes, morphological alterations of host cells, or the amplification of progeny phages (Al-Hindi et al., 2022). Importantly, such signal generation occurs naturally and spontaneously, without the need for complex processing steps. Detection of phage-derived signals can be achieved through various analytical methods, including colorimetric, fluorescent, imaging, and molecular techniques such as RT-PCR. Previous studies have evaluated this potential to detect the presence of live target bacteria based on the (a) expression of exogeneous proteins/enzymes in target bacteria and its release from lysed bacteria (Chen et al., 2016, 2017a,b; Hinkley et al., 2018; Tilton et al., 2019; Wisuthiphaet et al., 2019, 2021); (b) amplification of phage numbers after infection measured using traditional plaque assays and fluorescence measurements of phage particles using imaging (De Siqueira et al., 2003; Mido et al., 2018; Rames and Macdonald, 2019; Sullivan et al., 2013; Wisuthiphaet et al., 2022); and (c) measurement of amplified phage DNA using RT-PCR and also quantification of phage proteins using mass spectrometry (Banu et al., 2014; Garrido-Maestu et al., 2019; Kutin et al., 2009; Rees and Barr, 2017; Reiman et al., 2007; Stanley et al., 2007; Wang et al., 2016). Despite these developments, many of these assays still have limitations, including the need to modify phages for exogenous expression in target bacteria and challenges in detecting amplified nucleic acid following phage infection via RT-PCR or imaging-based measurements of phage amplification, due to the limited portability of the measurement tools.

The combination of bacteriophage and CRISPR-Cas technologies for nucleic acid detection can address some of these limitations, enabling the development of a highly specific, sensitive, and portable detection method. However, this would require adapting and optimizing CRISPR/Cas technologies for the detection of amplified phage genomic DNA, which is significantly larger and more complex than the short DNA or RNA targets conventionally detected using CRISPR/Cas systems. Furthermore, these short DNA or RNA sequences are generated by exogenous amplification technologies such as RT-PCR or LAMP assays (Li L. et al., 2019; Brandsma et al., 2020; Lee and Oh, 2022; Wang et al., 2021, 2022); thus, the nucleic acid samples are relatively pure and are significantly amplified above potential background noise from other nucleic acids. In contrast, the phage-based approach relies on endogenous amplification of lytic phage after infection of viable bacteria, and the DNA isolate of amplified phage genomic DNA may contain a range of interferences, including bacterial and food DNA and other potential contaminants. Thus, the objectives of this study were to (a) optimize the detection of purified phage genomic DNA using CRISPR/Cas to determine the analytical sensitivity of the measurement; (b) evaluate the specificity and sensitivity using the optimized conditions to detect crude isolate of phage DNA after infection and lysis of the target bacteria and (c) determine the translation of the optimized approach to detect inoculated bacteria in a spinach homogenate, simulating a wash water conditions in the fresh produce industry.

In this study, the T7 phage and *E. coli* were selected as a model system. In this approach, infection of viable *E. coli* with T7 phages results in phage amplification within 25–30 min, followed by their release upon bacterial lysis. This amplified phage genomic DNA was isolated and detected using CRISPR/Cas12a. Optimization of CRISPR/Cas12a reaction conditions for detecting amplified bacteriophage T7 genomic DNA was conducted by evaluating the role of the crRNA sequence, the Cas12a:crRNA ratio, and reaction temperature in detecting target T7 genomic DNA. The optimized approach using T7 phage and CRISPR/Cas was validated by detecting viable *E. coli* in TSB medium and by detecting inoculated *E. coli* in a spinach homogenate, simulating spinach wash water as a real-world application. In summary, this study evaluates a novel approach to detect viable bacteria by combining phage infection of viable bacteria, amplification of phage genomic DNA, and detection using CRISPR/Cas12a. This approach is distinct from the other approaches that have used CRISR/Cas12a system such as Specific High-Sensitivity Enzymatic Reporter Unlocking (SHERLOCK) platform, which combines recombinase polymerase amplification (RPA) with CRISPR/Cas detection (Kellner et al., 2019) and HOLMESv2, which uses Loop-Mediated Isothermal Amplification (LAMP) in conjunction with CRISPR/Cas12b for one-step isothermal detection (Li L. et al., 2019). In these previous applications, nucleic acids, after isolation from target microorganisms such as bacteria or viruses, are amplified using isothermal or non-isothermal methods to generate short DNA or RNA molecules for CRISPR/Cas detection. Overall, this research advances the detection of target bacteria by developing novel applications of phages and the CRISPR/Cas approach for detecting amplified genomic phage DNA, and establishes the sensitivity and specificity of this approach in model systems, including a testing scenario relevant to the fresh produce industry.

## 2 Materials and methods

### 2.1 Materials and reagents

Alt-R Cas12a (Cpf1) Ultra nuclease, Alt-R^®^ A.s. Cas12a crRNA 1 (5’-AT GCC TCT TGG GAG GAA GAG A-3’) and crRNA 2 (5’-AT ACG ACT CAC TAT AGG GAG A-3’), IDTE buffer pH 7.5, and fluorescence probe (/5HEX/TTTTTTT/3IABkFQ/) were purchased from Integrated DNA Technologies (San Diego, CA, United States). T7-phage DNA (39.9 kbp) was purchased from Boca Scientific (Dedham, MA, United States). UNEX buffer was purchased from Microbiologics (St. Cloud, MN United States). Cen HiBind^®^ DNA Mini Columns were purchased from Omega Bio-Tek Inc. (Norcross, GA, United States). Ambion^®^ Nuclease-free water was purchased from Thermo Fisher Scientific (Waltham, MA, United States). Chloroform (ACS reagent, ≥ 99.8%) was purchased from ACROS Organics (New Jersey, United States). Tryptic soy broth (TSB) and tryptic soy agar (TSA) were purchased from Sigma-Aldrich (St. Louis, MO, United States). Phosphate buffer solution (PBS) was purchased from Fisher Bioreagent (Fair Lawn, NJ, United States).

### 2.2 Bacterial strain and bacteriophage propagation

*E. coli* BL21 (ATCC BAA-1025) were obtained from American Type Culture Collection (ATCC, Manassas, VA, United States). The culture stored at −80°C in glycerol stock (TSB containing 15% (v/v) glycerol) was revived by inoculating 10% (v/v) in TSB and incubating at 37°C with 200-rpm constant shaking for 16 h. The overnight culture was then streaked onto a TSA plate and incubated at 37°C for 16 h. The culture plates were stored at 4°C for short-time storage.

T7 phages (#BAA-1025-B2) were obtained from ATCC. Phage propagation was carried out by mixing T7 with log-phase *E. coli* BL21 and incubating at 37°C for 15 min to allow adsorption. Infected bacterial cells were harvested by centrifugation at 16,100 × *g* for 10 min, resuspended in TSB, and incubated at 37°C with shaking at 200 rpm until complete lysis (no visible turbidity) was observed. Chloroform (20% v/v) was then added to the mixture, vortexed vigorously, and the mixture was kept on ice for 10 min. The mixture was then centrifuged at 5,000 × *g* for 10 min to separate the cell debris. The supernatant containing T7 phage was collected and washed three times by resuspending the pellet in sterile PBS followed by centrifugation at 16,100 × g for 10 min. The T7 phages were then resuspended in sterile PBS and stored at 4°C until use.

### 2.3 Spinach homogenate preparation

Spinach homogenate was selected as a model of complex fresh produce samples. Baby spinach leaves were purchased from a local grocery store. Spinach homogenate was prepared by blending 50 g of spinach with 500 mL of sterile water using a sanitized blender for 30 s at maximum speed, twice. The solution was centrifuged at 11,000 × *g* for 10 min to separate the large plant particles. The supernatant was collected and centrifuged again at the same speed. The resulting supernatant was collected as the spinach homogenate. The chemical oxygen demand (COD) of the spinach wash water was measured to be approximately 3,000 mg/L.

### 2.4 Optimization of the CRISPR/Cas12a reaction

Purified T7 phage DNA was used to optimize the conditions of the CRISPR/Cas12a reaction. A working solution of T7 DNA at a concentration of 10 μg/mL was prepared by mixing with 1 μL of 100 μM of MgCl_2_ (0.1% v/v), followed by heating at 95°C for 5 min and rapid cooling at 4°C for 5 min. Two crRNAs sequences were evaluated at a final concentration of 500 nM, combined with 250 nM of Cas12a protein to form a ribonucleoprotein (RNP) complex followed by detection of T7 DNA (10 μg/mL) at 50°C. The crRNA that exhibited higher fluorescence signal was selected for subsequent optimization.

In order to optimize the concentrations of the crRNA and Cas12a enzyme, various concentrations of both components were tested to form an RNP complex. A 5 μL of crRNA (final concentration of 250 and 500 nM) was combined with 8 μL of Cas12a enzyme (final concentration of 125, 250, and 500 nM) in a 384 well-plate. The mixture was gently pipetted up-down for proper mixing and incubated at room temperature (25°C) for 20 min to allow RNP complex formation. Next, a 5-μL of the thermally treated T7 phage DNA and 5-μL of the fluorescence probe (final concentration of 500 nM) were added to the RNP complex. The reaction mixture was incubated at various temperatures (25, 30, 50, and 60°C) to determine the optimal temperature. The fluorescence intensity was measured using a microplate reader TECAN SpectraFluor Plus (TECAN Austria GmbH, Grödig, Austria), with excitation at 530 nm and emission at 580 nm.

After establishing the optimal reaction conditions, the detection limit of T7 DNA was evaluated. T7 phage pure DNA was serial diluted using IDTE buffer pH 7.5 to final concentrations of 0, 0.1, 0.5, 1, 5, 10, and 100 μg/mL, followed by analysis under optimized CRISPR/Cas12a condition.

### 2.5 Detection of *E. coli* based on T7 phage amplification

**FIGURE 1:**
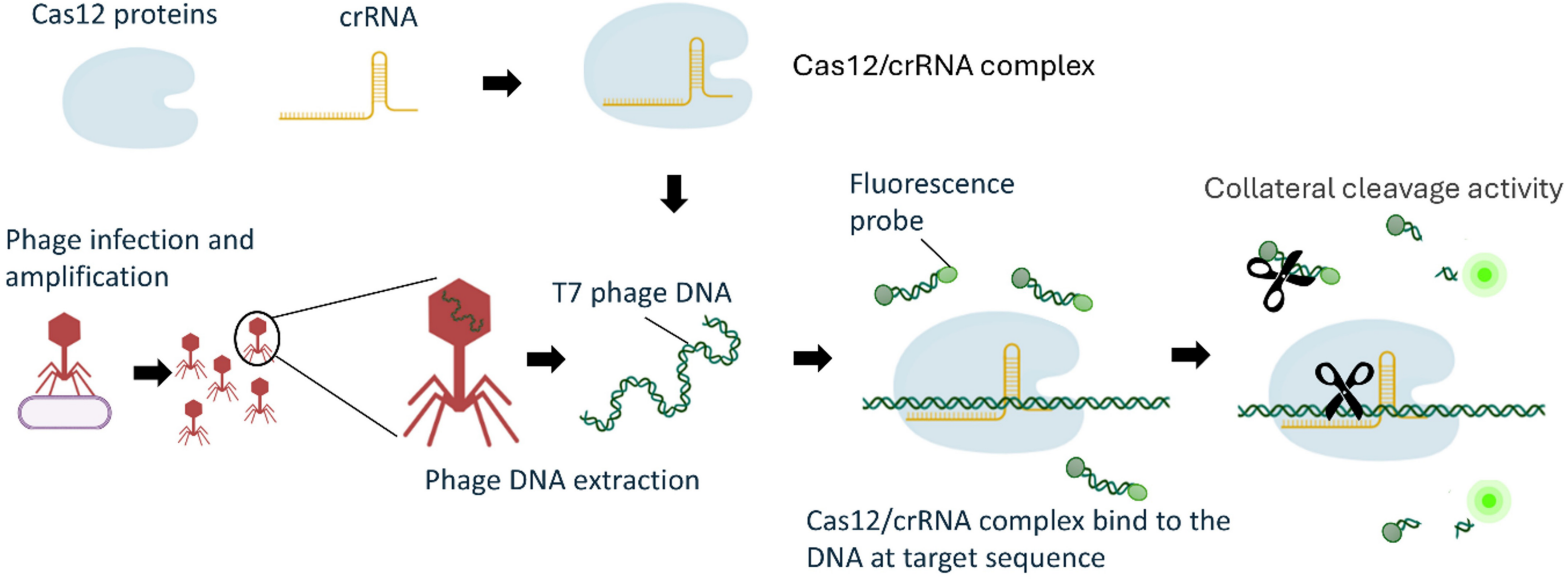
Schematic diagram of the phage amplification-coupled CRISPR/Cas12a system for selective detection of viable *E. coli.* Figure created with BioRender.com.

An overnight culture of *E. coli* BL21 was washed twice by centrifugation at 16,100 × *g*, followed by resuspension in sterile PBS. The bacterial suspension was then serially diluted in sterile PBS before inoculation into 10 mL of sterile TSB or a mixture of double-concentrated TSB and spinach homogenate to achieve the final concentrations of 0–10^4^ CFU/mL, and incubation at 37°C with 200 rpm constant shaking for enrichment. After, 4–5 h, T7 phage at a final concentration of 10^4^ PFU/mL of was added to the enriched culture and incubated under the same condition for 2 h for phage infection and amplification (see Figure 1 for a schematic overview).

To test the specificity of CRISPR/Cas12a-assisted phage system for the detection of viable *E. coli*, the CRISPR/Cas12a reaction under the optimized conditions was conducted using inactivated *E. coli*. To inactivate *E. coli* cells, 10^5^ CFU/mL of *E. coli* was incubated with 10% bleach for 10 min. The inactivated cells were washed twice before resuspension in sterile PBS. Serial dilution was performed to obtain 10^3^ CFU/mL of inactivated *E. coli*, which was enriched in TSB for 4 h, and then infected with T7 phage for 2 h.

To evaluate the specificity for the *E. coli* detection in mixed bacterial culture, the CRISPR/Cas12a reaction under optimized condition was used to detect *E. coli* with the presence of *Listeria innocua*, *Bacillus subtilis*, and *Staphylococcus epidermidis*. Each strain, at the initial concentration of 10^3^ CFU/mL, was mixed with an equal ratio (1:1:1:1). The mixture was enriched for 4 h, followed by infection with 10^4^ PFU/mL of T7 phage for 2 h.

A 500-μL aliquot of the T7 phage-infected *E. coli* solution was collected and mixed with 100 μL of 99% chloroform by vortexing, then incubated on ice for 5 min. The mixture was centrifuged the supernatant containing T7 phage particles was collected for detection using the optimized CRISPR/Cas12a assay.

### 2.6 T7 phage DNA extraction

A 200 μL aliquot of the T7 phage particles solution was mixed with 200 μL of UNEX buffer, vortexed thoroughly, and incubation at room temperature (25°C) for 10 min. Subsequently, 400 μL of absolute ethanol was added to the mixture and vortexed to ensure homogeneity. The mixture was transferred to a DNA Mini Column placed in a collection tube and centrifuged at 16,100 × *g* for 1 min. The flowthrough was discarded, and the column was washed twice with 500 μL of 70% ethanol, followed by centrifugation at 16,100 × *g* for 1 min. After discarding the flowthrough, the column was centrifuged again to remove any residual ethanol. For the DNA elution, the column was transferred to a sterile microcentrifuge tube, 100 μL of nuclease-free water was added to the column and subjected to centrifugation at 16,100 × *g* for 1 min. The flowthrough containing T7 phage DNA was collected for quantification using the optimized CRISPR/Cas12a reaction as previously described.

### 2.7 Statistical analysis

All experiments were performed in triplicate. Mean signal-to-noise (S/N) ratios and standard deviations were calculated for each group. Student’s *t*-test was performed to evaluate significant differences (α = 0.05) between the mean S/N values of two groups. For comparisons among multiple groups with different *E. coli* concentrations, Tukey’s honestly significant difference (HSD) test was applied (α = 0.05). All statistical analyses were conducted using Python (version 3.11.13) with the scipy, statsmodels, seaborn, and pandas libraries.

## 3 Results

### 3.1 Optimization of the CRISPR/Cas12a reaction and its detection limit

**FIGURE 2:**
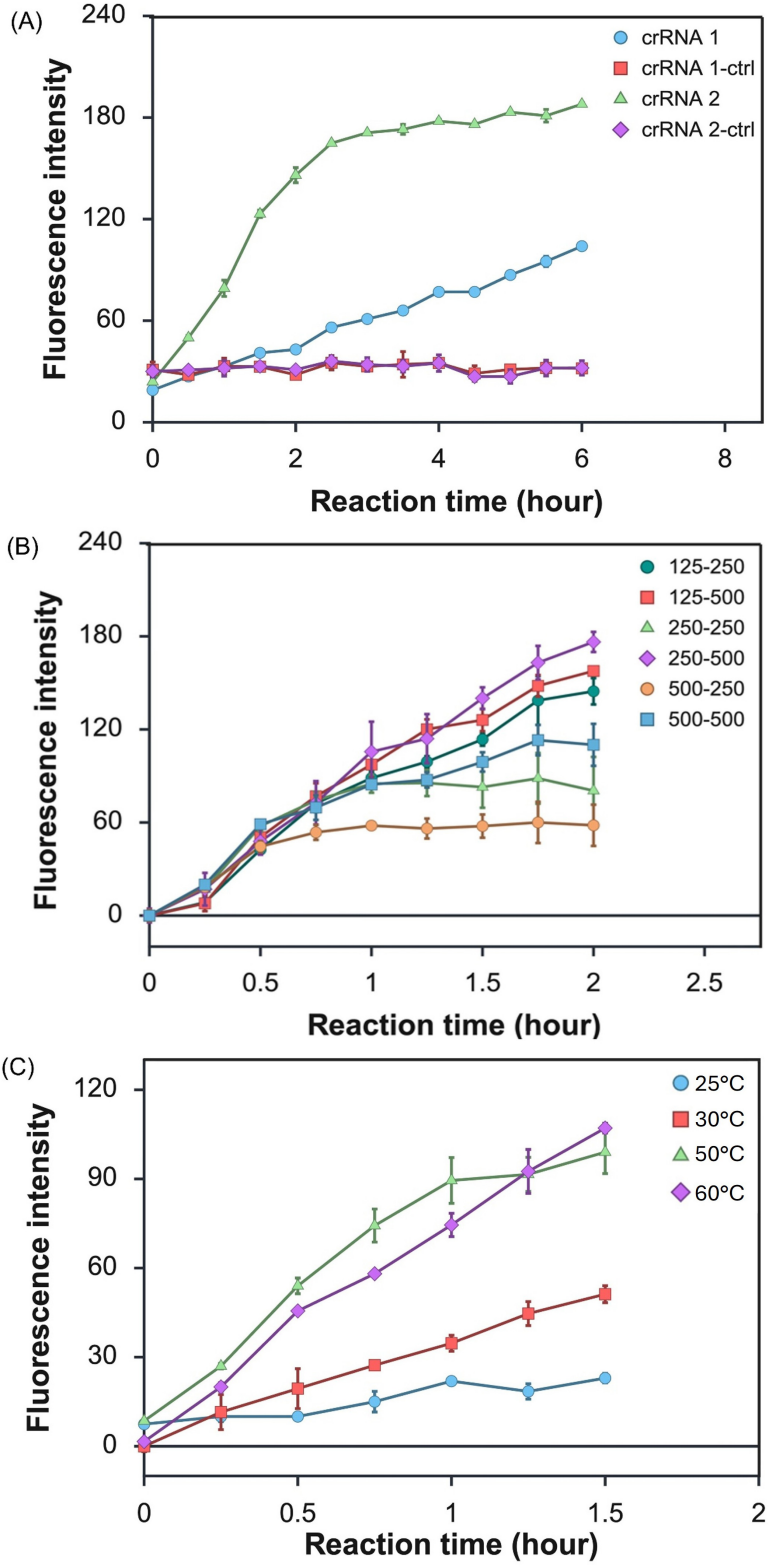
Time-course analysis of **(A)** the CRISPR/Cas12a reaction with T7 DNA using two different crRNA sequences, where crRNA1-ctrl and crRNA2-ctrl represent CRISPR/Cas12a reactions containing Cas12a and the corresponding crRNA without T7 DNA **(B)** CRISPR/Cas12a activity with target DNA based on the concentration levels of Cas12a at 125, 250, and 500 nM, and crRNA at 250 and 500 nM, respectively. The mixture of T7 DNA and CRISPR biochemicals was incubated at 50°C. **(C)** CRISPR/Cas12a activity with target DNA at 25, 30, 50, and 60°C. The reaction mixture contained Cas12a at a concentration of 250 nM and crRNA at 500 nM.

Two crRNAs were designed to target a specific region of the T7 phage genome adjacent to the protospacer adjacent motif (PAM) sequence, TTTV, where V is A, C, or G. The analytical performance of the two crRNAs was evaluated for their relative efficiency based on the increase in fluorescence intensity during the CRISPR/Cas 12a reaction. For the CRISPR/Cas12a reaction, the RNP complex was formed using 500 nM of a selected crRNA and 250 nM of Cas12a nuclease, followed by incubation of the complex with 10 μg/mL of T7 phage DNA at 50°C. The increase in fluorescence intensity generated by the CRISPR/Cas12a reaction was measured at 15-min intervals over a total of 6 h. As shown in Figure 2A, crRNA2 generated a significantly higher fluorescence signal, reaching the peak fluorescence intensity within 2.5 h of incubation. In contrast, crRNA1 produced a steadily increasing signal over 6 h that was lower compared to the peak fluorescence observed with crRNA2. The control samples without T7 phage DNA showed no significant change in fluorescence signal during the 6 h of incubation. The results indicate that the CRISPR/Cas12a reaction with crRNA2 showed higher sensitivity for detecting T7 DNA; therefore, crRNA2 was selected for further optimization of reaction parameters.

To determine the optimal concentration of Cas12a nuclease and crRNA for the CRISPR/Cas12a reaction, various concentrations of Cas12a nuclease (125, 250, and 500 nM) were tested at two levels of crRNA (250 and 500 nM). For these evaluations, the concentration of T7 phage DNA was fixed at 10 μg/mL, and the incubation temperature was maintained at 50°C. The results show that the rate of increase in fluorescence signal intensity was similar during the first 30 min across reactions with varying concentrations of Cas12a and crRNA (Figure 2B). After 30 min, the reaction with 500 nM Cas12a nuclease and 250 nM crRNA reached a plateau, suggesting limited cleavage of the reporter probe, possibly due to inefficient RNP complex formation. Overall, the levels of fluorescence signal achieved after 2 h of incubation were higher when the concentration ratios of Cas12a nuclease and crRNA were 1:2 and 1:4, and the strongest fluorescence signal was observed with 250 nM Cas12a and 500 nM crRNA. After 75 min, the increase in fluorescence was slower when Cas12a nuclease and crRNA concentration levels were 250 : 250 nM and 500 : 500 nM.

Reaction temperature is one of the factors that influence CRISPR/Cas12a reaction efficiency, particularly for relatively long DNA targets such as T7 phage DNA; thus, the reaction temperature was optimized by evaluating fluorescence signals under different conditions. Temperature optimization was performed using the previously optimized concentrations of Cas12a and crRNA (250 nM Cas12a and 500 nM crRNA) with 10 μg/mL T7 phage DNA. The influence of the incubation temperature was evaluated at 25, 30, 50, and 60°C. As shown in Figure 2C, reaction temperatures of 50 and 60°C resulted in a rapid increase in fluorescence intensity, suggesting higher reaction efficiency at these temperatures. Based on these results, 50°C was selected as the optimal reaction temperature for subsequent experiments.

**FIGURE 3:**
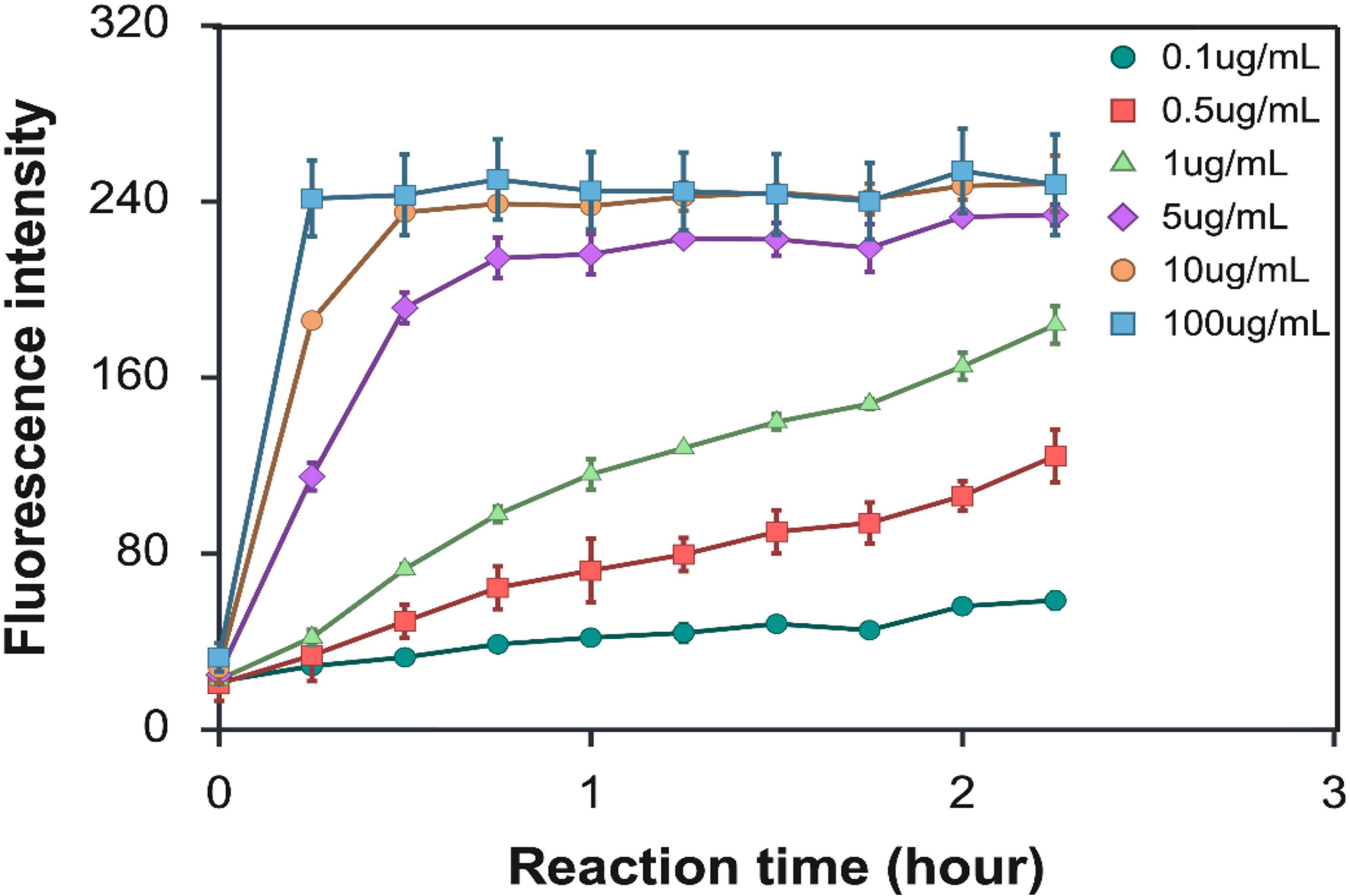
Time-course analysis of CRISPR/Cas12a activity with varying concentration levels of T7 DNA (0–100 μg/mL) at 50°C. The reaction mixture contained Cas12a 250 nM and crRNA 500 nM. Error bars indicate ± standard deviation of means.

The detection sensitivity of the optimized reaction conditions was investigated by varying the concentration levels of T7 phage DNA in the range of 0–100 μg/mL. Under the optimized conditions (250 nM Cas12a, 500 nM crRNA, 50°C), samples containing T7 phage DNA at concentrations of 5 μg/mL or higher exhibited a rapid increase in fluorescence signal, reaching the saturation point within the first 30 min of the reaction (Figure 3). For T7 phage DNA concentrations of 0.5 and 1 μg/mL, fluorescence increased steadily during 2 h of reaction, albeit at a lower rate compared to the higher concentration levels of T7 phage DNA. A limited increase in fluorescence was observed for the T7 phage DNA concentration of 0.1 μg/mL; however, this low level of T7 phage DNA was clearly detectable after 75 min of the reaction (Figure 3). These results indicate that this CRISPR/Cas12a assay can detect T7 phage DNA at concentration levels as low as 0.1 μg/mL with a reaction time of 75 min. The fluorescence intensity obtained after a 75-min reaction under optimized CRISPR conditions exhibited an excellent linear correlation (*R*^2^ = 0.99) with T7 DNA concentration in the range of 0–1 μg/mL (Supplementary Figure 1).

### 3.2 Application of CRISPR/Cas12a reaction in detection of *E. coli*

**FIGURE 4:**
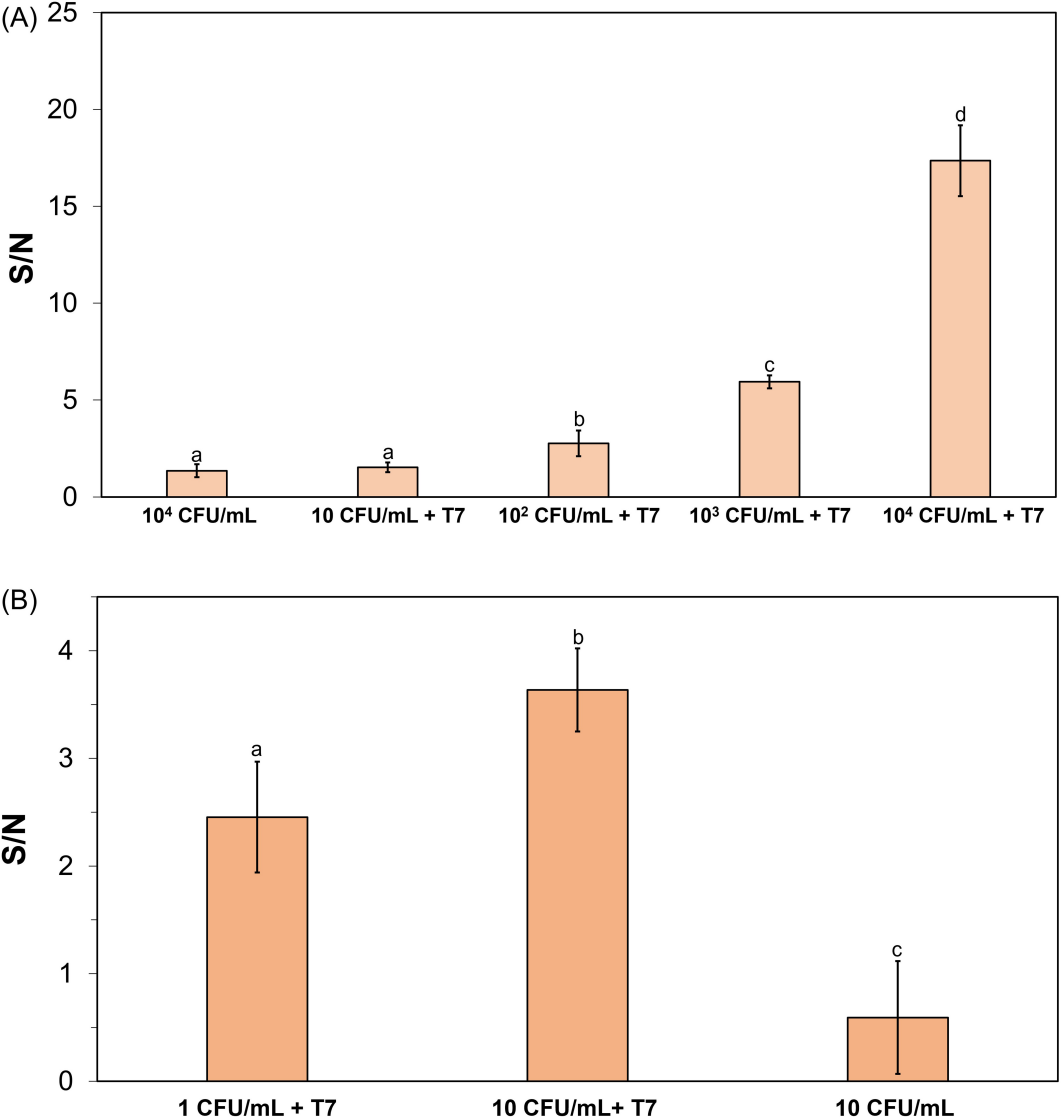
CRISPR/Cas12a detection of amplified T7 DNA extracted from T7 (10^4^ PFU/mL) after 2-h infection with **(A)** 4-h enriched *E. coli* BL21 (10–10^4^CFU/mL) and **(B)** 5-h enriched *E. coli* BL21 (1–10 CFU/mL). The reaction mixture contained Cas12a 250 nM and crRNA 500 nM and was incubated at 50°C. Treatments with different letters are significantly different (*p* < 0.05). Error bars indicate standard deviation of means.

To evaluate the applicability of the CRISPR/Cas12a assay for *E. coli* detection, the CRISPR reaction was used to detect amplified T7 phages resulting from the infection of *E. coli* with T7 phages. *E. coli* BL21 cells, at the initial concentration ranging from 1 to 10^4^CFU/mL, were enriched in TSB for 4–5 h before infection with T7 phage at 10^4^ PFU/mL for 2 h. The amplified T7 phages were isolated using solvent-induced bacterial cell lysis and precipitation of bacterial debris. The isolated T7 phage DNA was extracted using the approach described in the Methods section. The CRISPR reaction was performed under the previously optimized conditions to detect T7 phage DNA. Fluorescence measurements were represented as signal-to-noise (S/N) ratios, calculated by dividing the fluorescence signal from samples with amplified phages after infecting bacteria by the signal of the control sample (10^4^ PFU/mL T7 phage incubated in TSB for 2 h without *E. coli*). Figure 4A illustrates the S/N ratio of the fluorescence signal when 10–10^4^ CFU/mL *E. coli* BL21 were enriched for 4 h, followed by infection with 10^4^ PFU/mL T7 phages for 2 h, followed by 1 h of the CRISPR reaction under optimized conditions. The results indicate that the initial bacteria concentration levels at or above 10^2^ CFU/mL were detectable using this approach, as the S/N ratio was significantly higher than the negative control (10^4^ CFU/mL of *E. coli* without adding T7 phage). This negative control sample was also enriched and incubated under the same set of conditions, except without T7 phage infection. When the initial *E. coli* concentration was 10 CFU/mL, the S/N ratio was around 2; however, this was not significantly higher than the negative control sample of *E. coli* 10^4^ CFU/mL without T7 phages (S/N ∼1.4). To enhance detection sensitivity at lower bacterial loads (1 and 10 CFU/mL), the enrichment time was increased to 5 h prior to the phage infection step. After enrichment and 2-h T7 phage infection, both *E. coli* concentrations were detectable, with the S/N ratios significantly higher than the negative control of *E. coli* 10 CFU/mL without T7 infection, but with the same enrichment treatment (Figure 4B).

### 3.3 Evaluation of the potential false-positive detection of *E. coli*

False-positive results in bacterial detection may result in additional costs and time for result confirmation or recalling non-contaminated products. In order to investigate the potential false positives in *E. coli* detection, the optimized CRISPR/Cas12a-based detection system was applied to detect inactivated *E. coli* and *E. coli* in the presence of multiple non-target bacterial strains.

**FIGURE 5:**
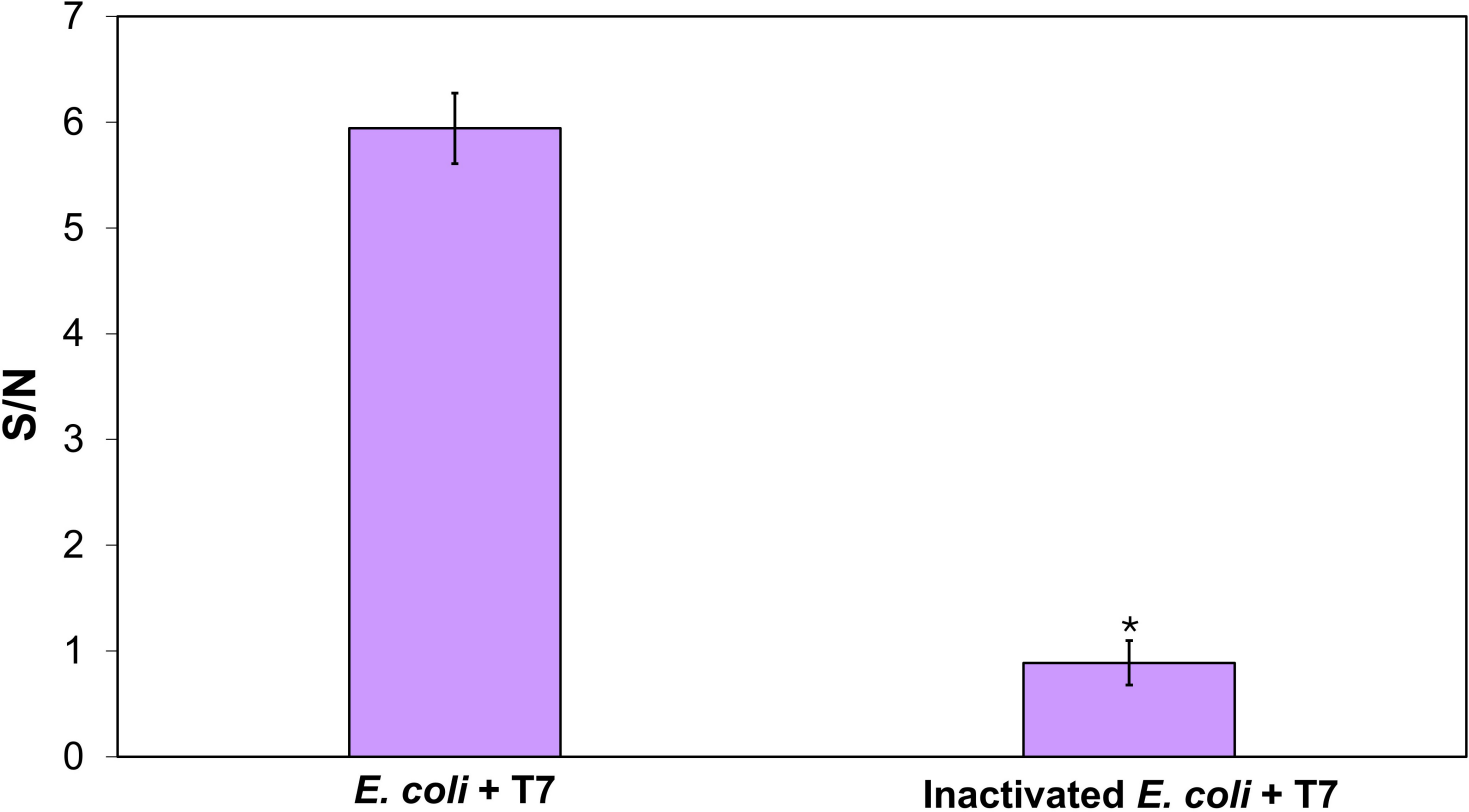
Determination of the detection specificity on viable *E. coli*. CRISPR/Cas12a detection of amplified T7 DNA extracted from T7 (10^4^ PFU/mL) after 2-h infection of target bacteria, following a 4-h enrichment of viable and inactivated *E. coli* BL21. The initial inoculum concentration was 10^3^CFU/mL. Treatments with “*” are significantly different (*p* < 0.05). Error bars indicate ± standard deviation of means.

*E. coli* cells were inactivated by treatment with 10% bleach for 10 min. The efficacy of the inactivation was confirmed by plating bleach-treated bacteria on TSA, where no bacterial growth was observed after overnight incubation. The inactivated *E. coli* at an initial concentration of 10^3^CFU/mL were enriched for 4 h, followed by T7 phage infection for 2 h and DNA extraction, before detection using the CRISPR/Cas12a reaction, mimicking the process adopted for viable cells. Figure 5 shows the S/N ratio obtained from inactivated *E. coli* compared to viable *E. coli* with T7 infection. The S/N ratio obtained from the inactivated *E. coli* was lower than 1 and significantly lower than those of viable *E. coli* (S/N ∼6). The results demonstrate the ability of this detection method to differentiate between viable and non-viable bacteria, thus highlighting the selectivity of the method.

**FIGURE 6:**
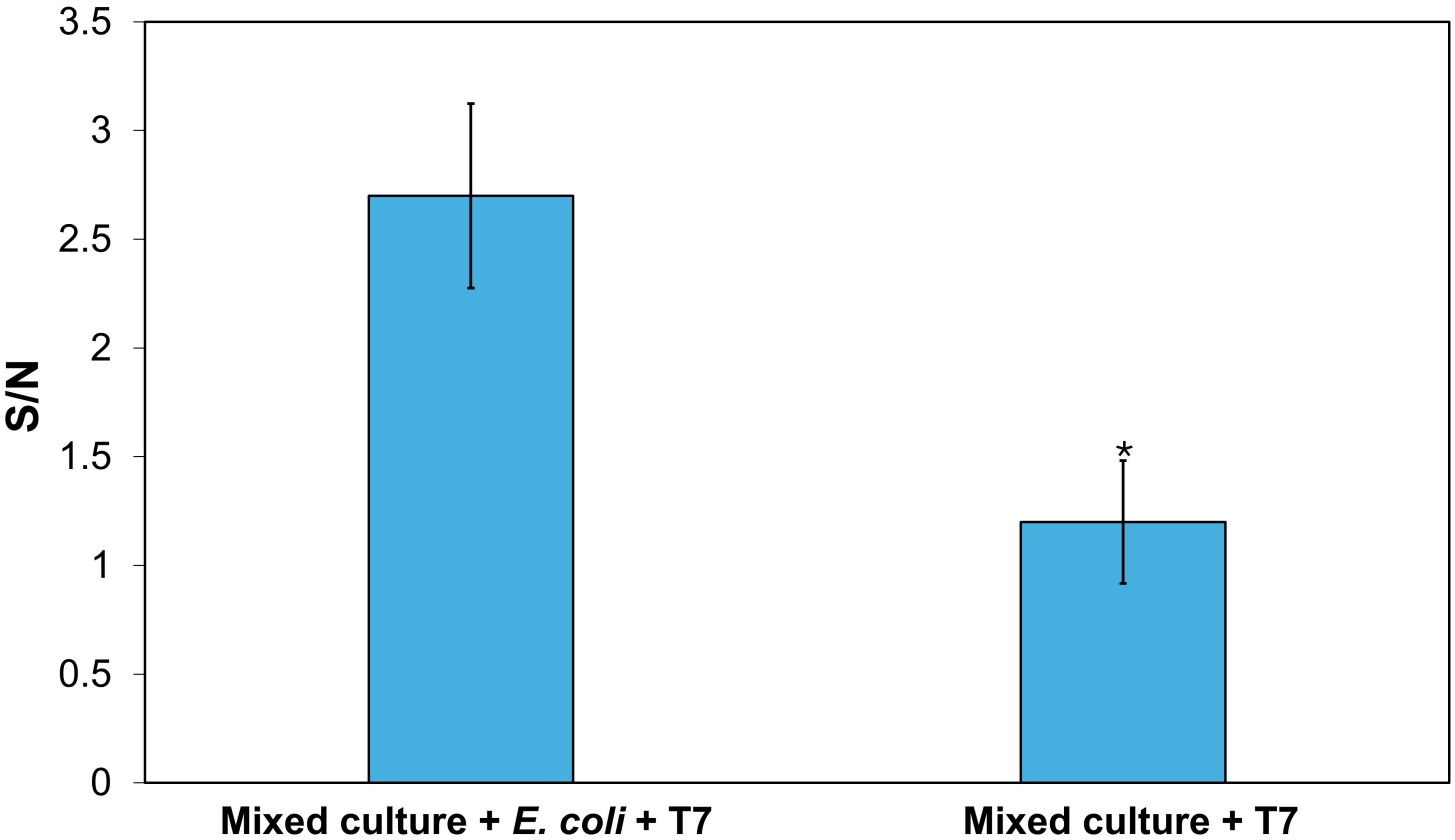
Determination of the detection specificity of target *E. coli*. CRISPR/Cas12a detection of amplified T7 DNA extracted from T7 (10^4^ PFU/mL) after 2-h infection of 4-h enriched 10^3^CFU/mL *E. coli* with the presence 10^3^ CFU/mL of *L. innocua*, *B. subtilis*, and *S. epidermidis*. Treatments with “*” are significantly different (*p* < 0.05). Error bars indicate ± standard deviation of means.

In food and environmental samples, there were other commensal bacteria that may interfere with the detection and potentially cause false-positive results. Therefore, the ability of the detection methods to selectively detect the target bacteria is critical. To evaluate the specificity of this detection method, *E. coli* BL21 was mixed with three non-target strains of bacteria, *L. innocua*, *B. subtilis*, and *S. epidermidis*. The initial concentration of all the bacterial strains was 10^3^CFU/mL. The mixed bacterial suspension was enriched for 4 h in TSB, followed by 2-h infection with T7 phage. DNA was then extracted and analyzed by the CRISPR/Cas12a reaction. The S/N ratio of *E. coli* detection in the mixed culture was 2.7, whereas the S/N ratio for the mixed culture without *E. coli* was 1.2 (Figure 6). These results demonstrate the high specificity of the detection approach in discriminating against background bacteria. It is important to note that the amplification of *E. coli* cells can be impacted in a mixed culture with other strains, as this may limit the efficiency of phage amplification.

### 3.4 Detection of *E. coli* in a model complex food matrix

**FIGURE 7:**
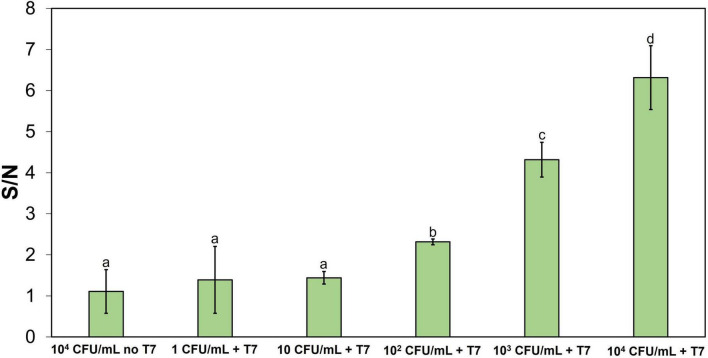
CRISPR/Cas12a detection of amplified T7 DNA extracted from T7 (10^4^ PFU/mL) after 2 h of infection of the 5-h enriched *E. coli* BL21 (1–10 CFU/mL) in a spinach homogenate. The reaction mixture contained Cas12a 250 nM and crRNA 500 nM and was incubated at 50°C. Treatments with different letters are significantly different (*p* < 0.05). Error bars indicate ± standard deviation of means.

Detection of bacteria in food samples can be challenging due to the presence of non-target bacteria and food debris resulting from homogenization of the food matrix. These factors can influence the amplification of target bacteria and the efficiency of bacteria-phage interactions, contribute to background fluorescence signal, and thus reduce the overall detection sensitivity. To evaluate the influence of these factors on the detection sensitivity, the analytical performance of the CRISPR/Cas12a detection system was evaluated using spinach homogenate as a representative complex food matrix. The spinach homogenate was selected as a model complex food matrix with high chlorophyll content and relatively high levels of commensal bacteria as it is a minimally processed product. Double-concentrated TSB was added to the spinach homogenate to support *E. coli* growth during enrichment. *E. coli* at 1–10^4^CFU/mL was inoculated in the spinach homogenate-TSB mixture and incubated at 37°C for 5 h, followed by incubation with 10^4^ PFU/mL T7 phage for 2 h. The fluorescence signal from the CRISPR reaction was measured after 1 h. The S/N ratio, calculated by dividing the fluorescence signal from spinach homogenate samples with amplified phages after bacterial infection by the signal from the control sample (10^4^ PFU/mL T7 phage incubated in spinach homogenate–TSB for 2 h without *E. coli*), are shown in Figure 7. The results show that at low initial concentrations (1 and 10 CFU/mL), the S/N ratio was 1.4, compared to 1.1 for the negative control (10^4^ CFU/mL of *E. coli* in the spinach homogenate without T7 phage infection). Increasing levels of the initial *E. coli* concentration to 10^2^, 10^3^, and 10^4^CFU/mL resulted in S/N ratios of 2.4, 4.3, and 6.3, respectively. These results indicate that *E. coli* at concentration of 10^2^ CFU/mL or higher can be detected in spinach homogenate with S/N ratio significantly above background.

## 4 Discussion

This study presents a specific detection method for detecting viable bacteria based on measuring bacteriophage DNA amplification upon the infection of target bacteria, using the CRISPR/Cas12a DNA detection system. CRISPR/Cas12a involves an RNA-guided nuclease that binds and cuts double-stranded target DNA, along with collateral cleavage on non-targeted single-stranded DNAs (Chen et al., 2018; Zhou et al., 2022). To enhance the analytical performance, several factors that influence the CRISPR/Cas12a reaction, including crRNA sequence, reaction temperature, Cas12a:crRNA ratio, were optimized.

The design of the crRNA plays a pivotal role in determining the kinetics of target recognition and signal activation (Kellner et al., 2019). The crRNA sequences in this study were selected based on the previous studies that had designed primers targeting specific regions of the T7 genome. crRNA1 was designed according to the primer sequence for qPCR quantification of T7 phage DNA (Peng et al., 2018), whereas crRNA2 was designed based on the primer designed to amplify a promoter sequence of T7 RNA Polymerase (Khateb et al., 2007). As shown in Figure 2A, crRNA2 produced a more rapid fluorescence signal increase compared to crRNA1, indicating faster reaction kinetics under identical reaction conditions. This observed difference could be attributed to the accessibility of the target sites (O’Brien et al., 2023). The promoter region targeted by crRNA2 is transcriptionally active, thereby facilitating more efficient Cas12a binding and activation. On the other hand, the region targeted by crRNA1, although suitable for qPCR quantification, may be structurally constrained or less accessible, leading to slower RNP complex formation and delayed signal generation (Zhou et al., 2024).

The ratio of Cas12a enzyme to crRNA is a key factor influencing the CRISPR reaction efficiency. An imbalanced ratio can lead to insufficient RNP complex formation or reduce collateral cleavage of the probes (Chenouard et al., 2023). In previous CRISPR/Cas12 application studies, reactions were performed with Cas12a:crRNA ratios close to 1:1, with the concentration ranging from 100 to 1,200 nM (Li L. et al., 2019; Hsieh et al., 2020; Ding et al., 2021; Kim et al., 2021; Zhou et al., 2022; Liu et al., 2022). However, our findings indicate that higher ratios (Cas12a: crRNA of 1:2 and 1:4) resulted in greater fluorescence signal intensity for the detection of T7 phage genomic DNA (Figure 2B). Consequently, the concentration of 250 nM Cas12a and 500 nM crRNA were selected as optimal for the detection of the target DNA.

The reaction temperature is a critical parameter that influences the analytical performance of the CRISPR/Cas12a reaction. In this study, the reactions at 50 and 60°C gave a significantly higher reaction rate compared to lower temperatures of 25–30°C (Figure 2C). These results are in agreement with findings from Li L. et al. (2019), which reported that the optimal temperature for Cas12b trans-cleavage within 45–55°C range (Li L. et al., 2019). Similarly, the optimal temperature for the one-pot CRISPR reaction for SARS-CoV-2 detection was reported to be 52°C (Ding et al., 2021). Although several previous CRISPR/Cas-based bacterial detection platforms reported a reaction temperature of 37°C (Liu et al., 2021; Wang et al., 2021; Wei et al., 2024; Zhu et al., 2023), 50°C was selected as the optimal reaction temperature for the detection of T7 phage DNA. Higher temperature likely promotes the unfolding of the DNA strand, especially for relatively large T7 genomic DNA molecules, thereby improving target accessibility for the Cas12a-crRNA complex.

The sensitivity of the T7 DNA detection using the optimized CRISPR/Cas12a reaction condition was evaluated with purified T7 DNA with a concentration range of 0–100 μg/mL. Results showed that 5 μg/mL of T7 DNA could be reliably detected within 30 min, whereas low concentrations of 0.1 μg/mL were detectable after 1 h of the reaction (Figure 3). CRISPR/Cas sensing technique is reported to have high sensitivity based on the detection of short target nucleic acids (∼50–100 base) at attomolar level (10^–18^M) levels (Chen et al., 2018; Li et al., 2018a). However, the studies that have adopted this technique for bacterial DNA detection have reported significantly lower sensitivity. For example, Wang et al. (2021) reported the detection limit of 0.9 pg/μL of *E. coli* DNA using RPA as a DNA amplification method prior to CRISPR/Cas12a reaction (Wang et al., 2021). Higher sensitivity was achieved as reported in the study by Liu et al. (2022), where 10^–4^ ng/μL of genomic *Salmonella* DNA was detected with RPA followed by CRISPR/Cas12a reaction (Liu et al., 2022).

In this study, the detection of T7 phage DNA showed lower sensitivity compared to the aforementioned studies. However, this study did not use a PCR or isothermal amplification prior to the CRISPR/Cas12a reaction; therefore, the DNA template for the CRISPR/Cas12a reaction was the entire T7 phage genome, which is approximately 40,000 bp in size, significantly larger than a typical RPA amplicon (100–200 bp). The large size of the entire T7 genome directly impacts the copy number of target sequences in 1 mL. At an equivalent DNA mass concentration of 1 μg/mL, the estimated copy number of a 40,000 bp T7 phage genome is around 2.28 × 10^10^ molecules/mL, whereas a 200 bp RPA amplicon with the same mass concentration contains around 4.56 × 10^12^ molecules/mL. Thus, the T7 genomic DNA has approximately a 200-fold lower molar concentration than a 200 bp DNA fragment for the same mass concentration, resulting in fewer target molecules available for CRISPR/Cas12a recognition. Since CRISPR/Cas12a detection relies on absolute copy number rather than total DNA mass, the reduced copy number likely contributes to the observed lower sensitivity in detection based on genomic DNA. Additionally, long genomic DNA can reduce target site accessibility due to its complex structure, steric hindrance, or inefficient RNP complex, as well as the increased likelihood of non-specific binding and background noise. To address these limitations, future studies could explore other detection strategies, such as electrochemical methods, which may offer greater signal-to-noise ratios, even in complex food matrices (El-Moghazy et al., 2022). Furthermore, multiplexed crRNA designs targeting multiple regions across the phage genome could enhance detection sensitivity by enabling simultaneous activation of multiple Cas12a complexes.

Despite the DNA detection limit is relatively higher than that reported in other studies (Aman et al., 2020; Wang et al., 2021), this detection approach demonstrated the ability to successfully detect *E. coli* at 1 CFU/mL within 8 h 15 min, including pre-enrichment (5 h), phage infection (2 h), DNA extraction (15 min), and CRISPR reaction (1 h). The sensitivity of this method was comparable to that reported by Zhu et al. (2023), which achieved a detection limit of 1 CFU/mL of *E. coli* using Recombinase-Aided Amplification (RAA) combined with CRISPR/Cas12a technology (Zhu et al., 2023), and similar to the study by Lee and Oh (2022), which reported a detection limit of 1.22 CFU/mL for *E. coli* using the LAMP-CRISPR/Cas12a system (Lee and Oh, 2022). However, the sensitivity of the method developed in this study was higher than those reported by Wang et al. (2021) which the detection limit of 6.5 × 10^4^ CFU/mL was obtained using RPA-CRISPR/Cas12a technique (Wang et al., 2021).

A key distinction of the present approach is its reliance on phage-mediated DNA amplification rather than direct amplification of bacterial DNA through PCR or isothermal methods. Here, the CRISPR/Cas12a assay targets T7 phage genomic DNA, which is amplified during the phage replication process upon infection of viable *E. coli* cells.

In addition, by using CRISPR/Cas12a, which targets and cleaves the DNA template, no transcription step is required before the detection reaction, making this method less complicated than other detection methods utilizing CRISPR/Cas13-based approaches that require transcription (Kellner et al., 2019). Furthermore, this study employed a simple DNA extraction method using UNEX buffer, eliminating the need for commercial DNA extraction kits. Conventional DNA extraction methods often require multiple purification steps, which can be time-consuming and may lead to DNA loss. On the other hand, the UNEX buffer enables efficient extraction of DNA in a single step, reducing both processing time and costs. This simplified approach enhances the potential of this approach for practical applications, particularly for rapid and on-site detection of foodborne pathogens.

Detection of specific bacteria based on the amplification of phages upon infection of the target bacteria has been investigated using various techniques. The most traditional method for detecting phage amplification is the use of culture-based methods, such as plaque assays; however, the extended incubation time required limits its suitability for rapid detection applications (Anderson et al., 2011). The fluorescence imaging method has been investigated as an alternative method, offering a simpler and faster procedure, with the detection of 10 CFU/mL of *E. coli* achievable within 8 h (Wisuthiphaet et al., 2022). To improve detection sensitivity, molecular-based techniques were used to monitor phage DNA amplification. For example, Luo et al. (2022) demonstrated the detection of *Acinetobacter baumannii* in swab samples using phage amplification and Taqman qPCR to detect amplified phage DNA, with a detection limit of 1 log CFU/mL. In this approach, the authors reported 3 h of co-culture with phages, followed by qPCR approach to detect amplified DNA (Luo et al., 2022). Choi et al. (2024) reported the development of phage T4-based colorimetric sensing material for *E. coli* detection in drinking water and ground beef, with a detection limit of 10^2^ CFU/mL of *E. coli* achieved within 8–9 h (Choi et al., 2024). In addition, Alonzo et al. (2022) reported an engineered phage based detection approach and a microfluidic device for *E. coli* detection in drinking water with a detection limit of ∼4 CFU/100 mL within 5.5 h. In this study, CRISPR/Cas12a-based DNA detection was utilized to detect amplified T7 phage DNA, with a detection sensitivity of 1 CFU/mL of *E. coli* in culture and 10^2^ CFU/mL in spinach within an 8-h timeframe, achieving detection levels similar to other phage-based detection of bacteria in complex samples (Choi et al., 2024; El-Moghazy et al., 2022; Wisuthiphaet et al., 2022). In contrast to the native phage-based detection approach, there are several genetically modified phage-based detection systems that achieved slightly higher detection sensitivity within a similar detection time frame, ranging from 5 to 8 h (Alonzo et al., 2022; Chen et al., 2017a; Duong et al., 2020; Wang et al., 2017, 2019). However, these approaches require phage genetic modification or reporter gene expression and often rely on methods such as microfluidics to improve the kinetics of phage-bacterial interactions and the detection signal. This study uses wild-type T7 phage, simplifying phage preparation and demonstrating the flexibility of wild-type phage for detecting target bacteria in food systems. Furthermore, integration of phage amplification with the CRISPR/Cas12 isothermal detection approach provides a novel direction to advance phage-based detection of bacteria beyond reporter genes with engineered phages and other resource-intensive approaches, including fluorescence microscopy and qPCR (Garrido-Maestu et al., 2019; Yang et al., 2020; Wisuthiphaet et al., 2022; Luo et al., 2022; Yi et al., 2023). The detection sensitivity of this approach can be potentially further enhanced by optimization of various parameters, such as crRNA sequence, number of crRNA, pre-treatment of the sample, and signal readout strategies, which could be systematically optimized to improve both sensitivity and speed.

To evaluate the real-world applicability of the developed detection system, the detection of *E. coli in* spinach homogenate as a complex model food matrix was performed. The results indicate that this detection approach was able to detect 10^2^ CFU/mL of *E. coli* within 8 h and 15 min. Detecting bacteria in food matrices can be challenging due to the complex composition of food and the presence of background microflora, which can interfere with the detection signal and reduce the sensitivity of the detection method. Therefore, pre-enrichment of bacteria before detection is essential. For this reason, the detection of *E. coli* in spinach homogenate requires an additional hour for pre-enrichment to detect 10^2^ CFU/mL of *E. coli*, resulting in a total detection time of 8 h 15 min. In contrast, the lower detection limit for *E. coli* in culture media is 1 CFU/mL, achieved within 8 h and 15 min.

This detection approach also offers a relatively simple setup compared to other molecular-based methods, as the CRISPR/Cas12a reaction is isothermal, thus there is no requirement for an advanced instrument. The lab components used in this study, including an incubator, vortexer, heat block, centrifuge, and fluorescence reader, are now available in compact, portable formats, enabling deployment outside centralized laboratories. Additionally, recent advances in field-deployable laboratory devices further enhance the feasibility of applying this platform in on-site food safety testing in resource-limited settings (Bhupathi and Devarapu, 2021; Shin et al., 2021; Wight et al., 2020). While CRISPR/Cas12a combined with DNA amplification has been applied for the detection of bacteria in food samples, primarily in milk, juice, and meat products (Selvam et al., 2022), its application in fresh produce consumed raw, such as lettuce and spinach, remains limited. One of the challenges associated with such matrices is that the natural fluorophore, such as chlorophyll, can increase the background fluorescence signal, thereby limiting detection sensitivity. Also, the presence of the food matrix may interfere with phage-bacterial interactions.

Table 1 provides a summary of the detection limits reported in other studies that used CRISPR/Cas12a for *E. coli* detection in various complex food matrices. The table also shows a broad range of detection sensitivities, with the lowest being 1 CFU/mL in skim milk and drinking water, and the highest at 14 CFU/mL in ground beef. Most studies achieved these detection limits by incorporating a pre-amplification step, using methods such as Recombinase Polymerase Amplification (RPA), Multienzyme Isothermal Rapid Amplification (MIRA), or Entropy-Driven Amplification Reaction (EDC).

**TABLE 1:** Summary of CRISPR/Cas12a-based *E. coli* detection in complex food matrices.

Sample matrix	Pre-amplification method	Limit of detection (LOD)	References
Meat products	Recombinase polymerase amplification (RPA)	10 Copies of target gene	(Liu et al., 2021)
Ground beef	Multienzyme Isothermal rapid amplification (MIRA)	14 CFU/mL	(Wang et al., 2021)
Romain lettuce	Looped mediated isothermal amplification (LAMP)	4.8 CFU/g	(Lee and Oh, 2022)
Skim milk and drinking water	Recombinase-aided amplification (RAA)	1 CFU/mL (fluorescence method) 10^2^ CFU/mL (lateral flow assay)	(Zhu et al., 2023)
Water and milk	Entropy-driven amplification reaction (EDC)	5.02 CFU/mL	(Cui et al., 2024)
Milk	Recombinase polymerase amplification (RPA)	1 CFU/mL	(Song et al., 2024)
Water and milk	Entropy-driven catalysis (EDC)	5 CFU/mL	(Huang et al., 2025)
Romaine lettuce	Recombinase polymerase amplification (RPA)	10 CFU/g	(Lee and Oh, 2025)
Culture broth	Phage amplification	10 CFU/mL	This study
Spinach homogenate	Phage amplification	10^2^ CFU/mL	This study

One of the key advantages of using phage amplification is the ability to discriminate between viable and non-viable cells, as only viable cells can be infected by phages, resulting in phage amplification. As shown in Figure 5, the inactivated *E. coli* yielded an S/N ratio lower than 1, confirming that this detection approach minimizes false-positive results from non-viable cells. This discrimination is vital for food processing, as one of the key goals of the food process is to inactivate bacteria in food products and contact surfaces. Consequently, nucleic acid-based detection methods are often complemented by culture-based methods to validate the results of molecular detection technologies (Kosai et al., 2021; Feng et al., 2011). However, such combined approach significantly extends the overall detection time (∼24–72 h), limiting the speed advantage of molecular detection methods. Another challenge in detection of bacteria in food matrices is the presence of background microflora, which can interfere with the detection signal and reduce sensitivity. In this study, a high specificity in the detection of target bacteria in the presence of mixed bacterial culture was demonstrated using bacteriophage in combination with CRISPR/Cas12a approaches (Figure 6).

Despite its advantages, this proposed method still has some limitations. First, the assay requires an enrichment step, which extends the overall detection time. However, enrichment remains necessary for detecting viable cells, as it supports the resuscitation of injured viable cells, enhances bacterial counts, and potentially revives metabolically inactive bacteria isolated from food systems. Therefore, enrichment is beneficial for enabling efficient phage infection, and amplification is most active in metabolically active log-phase bacteria. Future efforts to minimize the enrichment time could be achieved by isolating or concentrating the target bacteria prior to enrichment. In addition, optimization of growth media, including the use of selective media, may help reduce the growth of endogenous microflora while supporting the growth of target bacteria. Second, this study employed *E. coli* BL21 as a model strain to demonstrate the overall concept, as the *E. coli* BL21-T7 phage system is well characterized and widely used for developing phage-based biosensors (Chen et al., 2015, 2017a,b, 2021; Hinkley et al., 2018; El-Moghazy et al., 2022). However, *E. coli* BL21 does not fully represent the diversity of foodborne pathogenic *E. coli* strains. Therefore, future studies focused on optimizing phage strains for infecting a range of foodborne and pathogenic *E. coli* strains will enable the translation of the approach into real-life scenarios. Recent advances in CRISPR-based detection also offer opportunities to improve this approach. For example, the PAM-less exonuclease-assisted Cas12A nucleic-acid detection, which enables flexibility in crRNA design and supports low-cost, instrument-free, visual readouts (Ng et al., 2025). Integrating PAM-less Cas12 technology with the phage-amplification approach could further improve the applicability and robustness of the phage amplification-CRISPR/Cas12a system.

Overall, this study demonstrates the successful integration of bacteriophage amplification with CRISPR/Cas12a-based DNA detection for the rapid and specific detection of *E. coli*. By implementing phage infection, this assay selectively targets viable bacteria without the need for additional DNA amplification steps. This approach achieved a detection limit of 1 CFU/mL of *E. coli* in culture broth and 10^2^ CFU/mL in a complex food matrix within 8 h 15 min, highlighting its applicability for food safety monitoring that supports same-day decision making. Furthermore, its isothermal nature, simple DNA extraction process, and minimal instrumentation requirements make this approach well-suited for point-of-need bacterial detection in field settings. Notably, the utilization of bacteriophages offers a versatile platform for future optimization, as both detection time and host range can be improved through genetic engineering approach (Burke et al., 2025; Zhang et al., 2025). This approach also allows for the target detection of specific serotypes, such as *E. coli* O157:H7 with specific phage (Zhou et al., 2023), and the differentiation of viable pathogens from non-pathogenic species such as the detection of *Listeria monocytogenes* and its differentiation from *Listeria innocua* (Akhtar et al., 2017; Zolti et al., 2022). By targeting only live cells, this method effectively mitigates the risk of false-positive results from residual DNA after the sanitation process, thereby reducing the need for isolation, serotyping, or genetic confirmation steps (Panhwar et al., 2024; Olszewska et al., 2025). Furthermore, this platform shows potential for broader functional applications, including antibiotic susceptibility testing (Sorokulova et al., 2014). Future research should explore strategies to enhance sensitivity, particularly for large genomic DNA targets, and to expand applicability to other bacterial pathogens and diverse food matrices.

## 5 Conclusion

This study establishes a novel detection strategy that combines bacteriophage amplification with CRISPR/Cas12a-based detection for the rapid and specific detection of viable *E. coli*. Under optimized reaction conditions, this approach successfully detected as low as 1 CFU/mL of *E. coli* in culture broth and 10^2^ CFU/mL in spinach homogenate within 8 h 15 min. By utilizing the rapid replication cycle and host specificity of bacteriophages, this detection approach enables selective targeting of viable bacterial cells, without the need for traditional nucleic acid amplification, as only live bacteria facilitate phage replication and amplification of phage DNA. This approach also demonstrated high specificity toward target bacteria regardless of the presence of a mixture of non-target bacteria and complex food matrices. Moreover, this detection approach requires isothermal detection of the amplified phage DNA using CRISPR/Cas12a, which can be achieved through a simple procedure, supporting its potential for adaptation into rapid, on-site detection platforms.

## Data Availability

The original contributions presented in this study are included in the article/Supplementary material, further inquiries can be directed to the corresponding author.
