# Supplementary material for: Phage amplification-coupled CRISPR/Cas12a system for selective detection of viable E. coli in fresh produce
Source: Front Microbiol. 2026 Apr 1;17:1770383. doi: 10.3389/fmicb.2026.1770383 (PMC13079353; doi:10.3389/fmicb.2026.1770383)
Supplement: Supplementary file 1 [file Data_Sheet_1.docx]

**Figure S1** The relationship between the quantity of T7 phage DNA (µg/mL) and the relative fluorescence intensity after a 75-minute CRISPR-Cas12a reaction.
